# Arthroscopically assisted refixation of bony ACL tears with accompanying posteraleral tibial plateau (“apple bite”) fractures – a minimally invasive treatment approach

**DOI:** 10.1007/s00068-026-03122-7

**Published:** 2026-03-03

**Authors:** Thomas Rosteius, Ole Somberg, Maria Bernstorff, Sebastian Lotzien, Jan Gessmann, Thomas Armin Schildhauer, Matthias Königshausen

**Affiliations:** Department of General and Trauma Surgery, BG University Hospital Bergmannsheil, Bürkle- de- la- Camp Platz 1, 44789 Bochum, Germany

**Keywords:** Tibial plateau fracture, Articular congruity, Apple bite fracture, Eminence fracture

## Abstract

**Purpose:**

Combined fractures involving bony avulsion of the anterior cruciate ligament (ACL) with a concomitant impression fracture of the posterolateral tibial plateau are rare injuries, with limited data available in the literature. Therefore, the aim of this study was to evaluate the functional and clinical outcomes of arthroscopically assisted treatment for these osteoligamentous injuries.

**Methods:**

We retrospectively reviewed 16 patients after a mean follow-up of 24.3 ± 10.6 months (12–45 months) who underwent arthroscopically assisted treatment for these named injuries. The fixation of the ACL avulsion was carried out either with two crossed, cannulated 2.7 mm screws or using transosseous sutures (12 and 4 patients, respectively). The reduction and fixation of the tibial plateau fracture with an articular step-off greater than 2 mm was performed arthroscopically assisted by screw osteosyntheses (9 patients). Primary outcome parameters were the Lysholm score, Knee Injury and Osteoarthritis Outcome Score (KOOS), Numeric Rating Scale (NRS) Pain Score, and International Knee Documentation Committee (IKDC) score. Secondary outcome parameters included bony consolidation, complications, and surgical revisions.

**Results:**

The mean Lysholm score, KOOS and IKDC score were 84 ± 13, 81 ± 14%, and 78 ± 11, respectively. The NRS score had a median of 1.6, the median Tegner activity score was 4.5. Complete bony healing was achieved in all patients. Neither perioperative complications nor surgical revisions occurred. During follow-up, 6 patients showed persistent 1° laxity of the ACL in a side-to-side comparison without rotational instability. Two patients had a 5° extension deficit on the affected side.

**Conclusion:**

Arthroscopically assisted fixation of combined bony ACL tears and posterolateral tibial plateau fractures as a minimally invasive procedure results in good functional outcomes with sufficient joint stability. It offers the advantage of useful visualization of the joint surface, which helps to avoid residual intraarticular step-offs and posterolateral malalignment. ACL avulsion fixation is possible both through crossed screw osteosynthesis and transosseous sutures techniques in an arthroscopic setting.

## Introduction

Although tibial plateau fractures account for only 1% of all fractures [1, 2], these fractures are one of the most severe and challenging injuries of the knee joint. This is partly because it is a complex articular surface injury with very heterogenic fracture morphology, especially in the case of Arbeitsgemeinschaft für Osteosynthesefragen/ Orthopaedic Trauma Association (AO/OTA) type C fractures. Furthermore, in addition to different tibial plateau fractures, accompanying ligament injuries occur, which, if left untreated, can lead to chronic joint instability. This fact makes osteoligamentous injuries very specific and requires, in addition to fracture treatment, also ligament surgery. One of these rare injuries, that are caused by flexion/valgus force, are the examined eminence fractures with accompanying posterolateral impression. These injuries pose a significant challenge for orthopedic surgeons and the restoration of articular congruity, since the posterolateral corner of the tibial plateau can hardly be visualized despite the use of intraoperative fluoroscopy [3], hence its being called the “dark side of the knee” [4]. The lack of intraoperative visualization is one of the main reasons for inadequate reduction after complex tibial plateau fractures [5]. This leads to residual step-offs greater than 2 mm in up to 32% of all complex tibial plateau fractures [6]. A dislocated eminence fracture additionally carries the risk of persistent ACL- and rotational instability, making refixation absolutely necessary. Therefore, since eminence fractures are ideally treated arthroscopically [7], an arthroscopic approach is most suitable for the management of these combined injuries. This allows the stabilization of the eminence fracture and the direct visualization, reduction, and percutaneous fixation of the posterolateral joint surface.

Due to the rarity of these osteoligamentous injuries, the literature contains neither a gold standard nor targeted treatment strategy. Therefore, in this study, we analyzed the largest cohort of these rare fracture entity to date, treated entirely arthroscopically, in terms of short- to midterm outcome, complications, and surgical revisions.

We hypothesize that these combined injuries can be optimally treated arthroscopically, with low complication rates and very good functional outcomes.

## Materials and methods

The study was reviewed and approved by the local Institutional Review Board (IRB) (registered number: 18-6508_1-BR). All procedures were performed in accordance with the ethical standards of the institutional research committee and with the 1964 Declaration of Helsinki and its later amendments.

### Study design

Patients with combined bony ACL tear and posterolateral tibial plateau fracture between 2016 and 2020 were retrospectively reviewed. All patients with a minimum follow-up of one year were included in this study. Exclusion criteria were patients with accompanying collateral ligament or meniscal injury, vascular damage or previous damage of the joint and missing pre- and postsurgical computed tomography (CT) data. In total, 20 patients fulfilled these criteria. Sixteen out of these 20 patients were available for clinical follow-up and were considered for further analysis.

### Surgical management and postoperative procedures

Our guidelines for addressing bony ACL avulsions (1) and posterolateral fractures (2) were as follows:

1. Bony ACL avulsions were fixed if clinical anterior instability was present. Small, multi-fragment eminence fractures were more likely to be treated with transosseous sutures, whereas larger/ single bony eminence fragments were stabilized with cannulated screws.
2. The accompanying posterolateral fractures were reduced and stabilized if (1) there was an intra-articular joint depression greater than 2 mm and (2) the affected area extended more than 50% beyond the posterior horn of the lateral meniscus. Only small posterolateral rim fractures affecting less than 50% of the lateral meniscus were left untreated, regardless of the joint step.

**Fig. 1:**
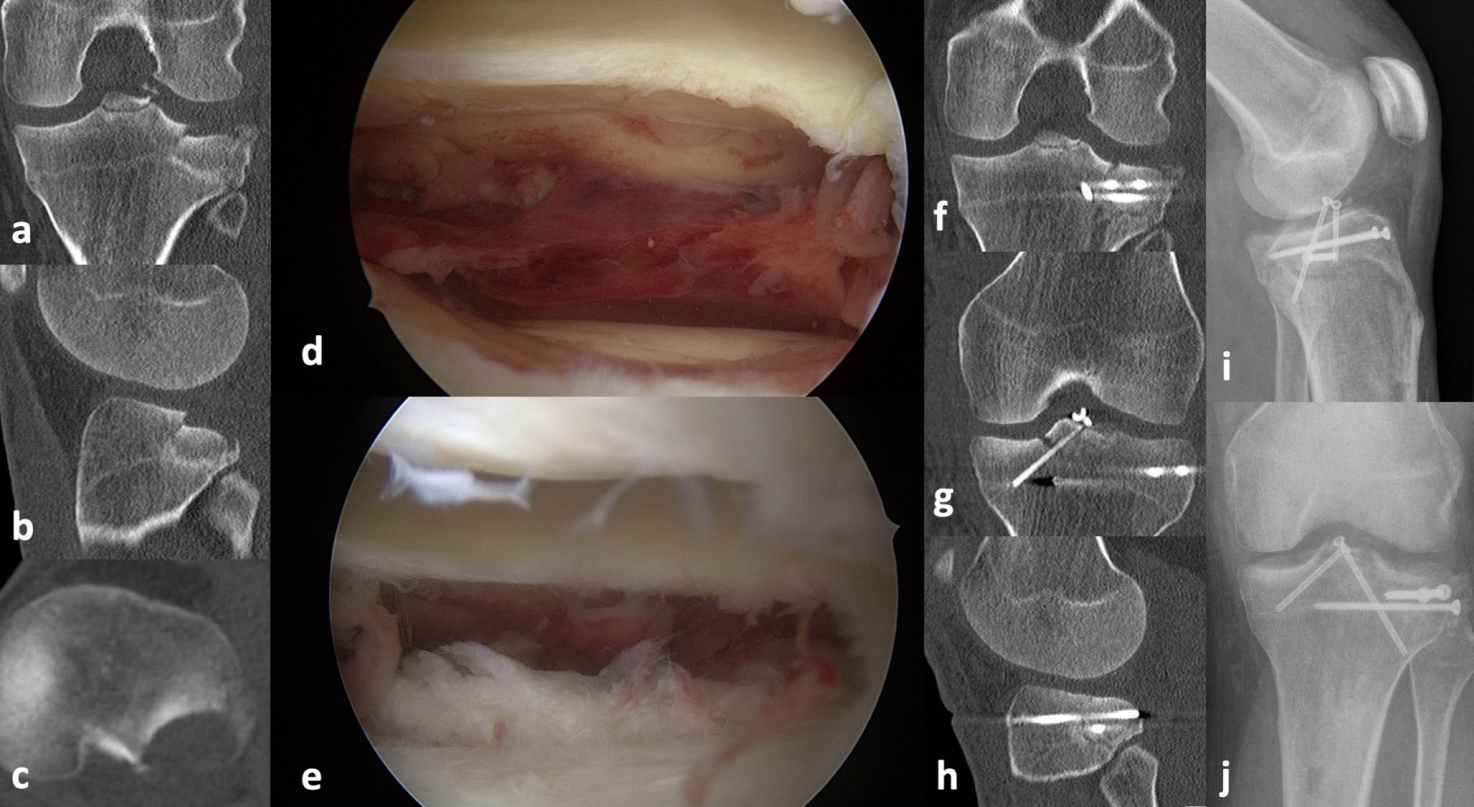
Thirty-nine-year-old patient with a Meyers-McKeever 2 eminence fracture and a Menzdorf type 2c posterolateral tibial plateau fracture (**a**, **b**, **c**). (**d**) and (**e**) demonstrate the initial arthroscopic view of the fracture and after reduction. (**f**, **g** and **h**) show the postoperative CT and (**i**) and (**j**) the final follow-up with complete bone healing

**Fig. 2:**
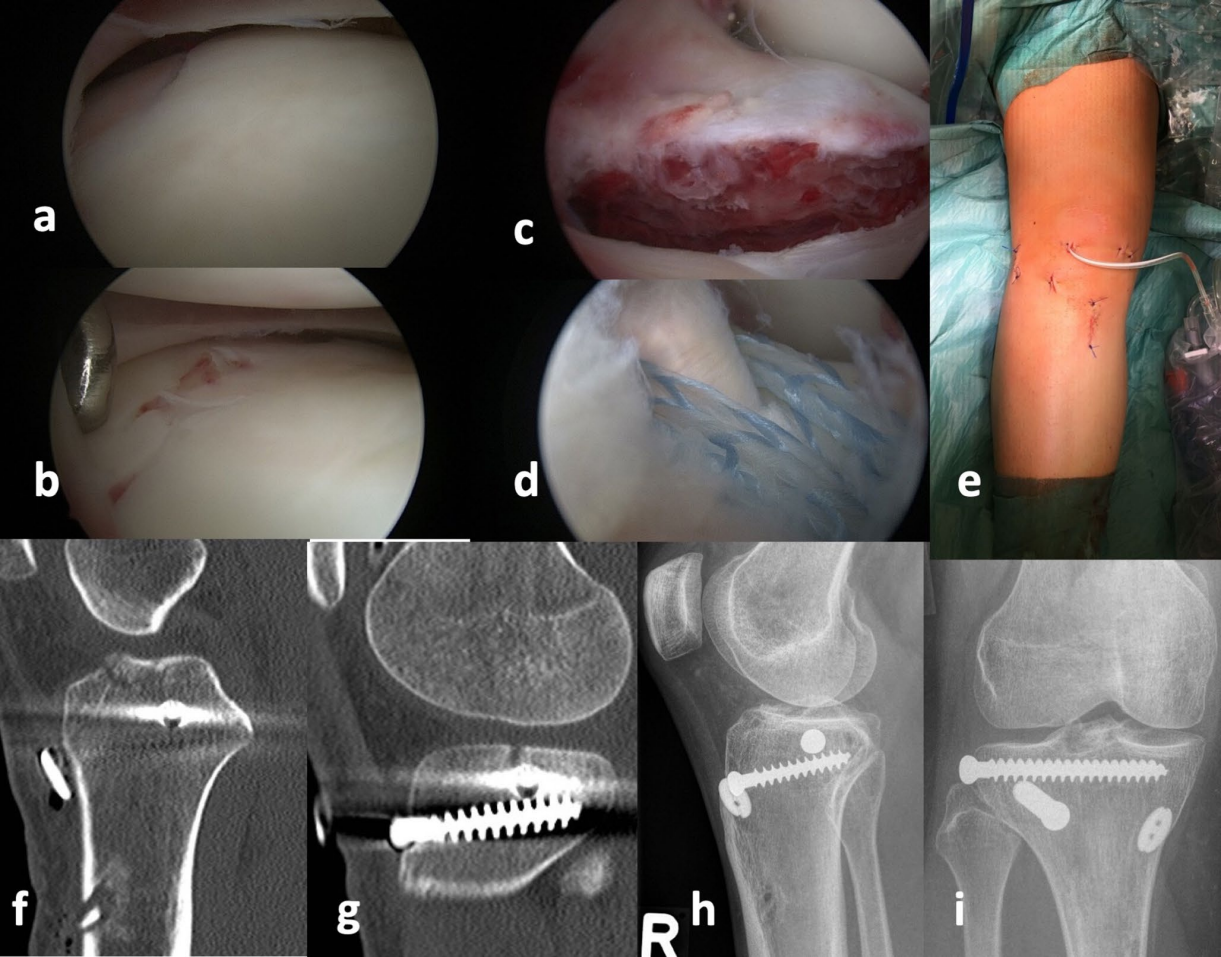
Posterolateral impression with accompanying dislocated eminence fracture. (**a** and **b**) demonstrate the initial impression and after reduction. (**c**) shows the dislocated bony avulsion of the ACL and (**d**) the fixation by using transosseous sutures. (**e**) demonstrates the less invasive treatment approach with minimal soft tissue compromise. Computed tomography was performed for postsurgical fracture position (f,** g**). X- rays at final follow up with complete fracture healing (**h**,** i**)

The bony ACL tears were fixed using either two crossed, cannulated 2.7 mm cortical screws (Fig. 1) or transosseous sutures (Fig. 2). The posterolateral tibial plateau fracture was reduced with the help of a cancellous bone ram and fixed using percutaneously inserted screw osteosyntheses after arthroscopic visualization according to the description of Ackermann et al. [8]. One patient needed additional posterolateral buttress plating due to a posterolateral shear fracture.

Physical therapy started 48 h after the operation with passive motion of the joint through a limited range- of- motion (ROM) (ex./flex. 0°/0°/90°) with the patient in the supine position. If necessary, peripheral nerve block anesthesia was applied. Patients had limited weightbearing (20 kg) and limited ROM for 6 weeks.

### Follow-up examination

The patient assessment and clinical evaluation were scheduled a minimum of one year after the primary surgery. Primary outcome parameters were the Lysholm score [9], Knee Injury and Osteoarthritis Outcome Score (KOOS), NRS Pain Score and IKDC Score [10]. Secondary outcome parameters were the assessment of bony consolidation, complications and revisions. Lower leg radiographs and CT images were obtained during the first 72 h after surgery to analyze the axial alignment and postsurgical articular congruity.

The ROM was measured using a goniometer.

### Statistical analysis

Descriptive data are described by the mean, standard deviation, minimum and maximum. After the normality of the data was tested using the Shapiro-Wilk test, normally distributed variables were assessed using the two-tailed t-test. Nonnormally distributed variables were analyzed with the Wilcoxon/Mann-Whitney test. Nominally scaled variables were compared using cross tables and Fischer’s exact test. α = 0.05 or less was considered statistically significant.

## Results

A total of 16 patients (36 ± 16 years old) with a mean/median follow-up of 24.3/ 24 ± 10.6 months (12–45 months) were included in the study. Table 1 shows the demographic data of the study group.

**Table 1:** Demographic data of the study group

Demographic data	
**n**	16
**Age (years) (mean ± SD)**	36.4 ± 16.4 (median = 34.5)
**Gender men / women (n)**	9 / 7
**Body mass index (BMI) (kg/m2)**	24.5 ± 5.4
**American Society of Anesthesiologists Physical Status Classification (ASA Score)**	
I II III IV	5 (31.25%) 8 (50%) 2 (12.5%) 1 (6.25%)
**Comorbidities (n)**	
No diseases < 3 diseases > 3 diseases	3 (18.75%) 9 (56.25%) 4 (25%)
**Meyers-McKeever classification**	
Grade I Grade II Grade III Grade IV	3 (18.75%) 5 (31.25%) 7 (43.75%) 1(6.25%)
**Menzdorf et al. classification**	
Ia Ib Ic IIa IIb IIc IIIa IIIb	3 (18.75%) 2 (12.5%) 0 3 (18.75%) 2 (12.5%) 5 (31.25%) 0 1 (6.25%)

In summary, one patient had a type 4 injury according to Meyers and McKeever, 7 patients a type 3 injury, 5 patients a type 2 and 3 patients a type 1 injury. All patients had an accompanying impression fracture of the posterolateral tibial plateau, in 2 patients in combination with a lateral split component and in 2 patients with a medial split. In 12 patients, the bony ACL tears were fixed using crossed, cannulated screws and in 4 patients using pull-out sutures. In 9 patients, the accompanying tibial plateau impression fracture was reduced arthroscopically assisted and fixed percutaneously; in 7 cases, the posterolateral fracture was not surgically addressed. Table 2 demonstrates the final outcome and complications. Complete bone healing was achieved in all patients. There were neither perioperative complications nor surgical revisions, including no ACL reconstructions during follow-up. In 7/16 patients (7/9 patients with fixation of the posterolateral fracture), a complete implant removal was performed. Six patients showed a remaining 1° laxity of the ACL in comparison to the other side. Two patients had an extension deficit of 5° on the affected side. No rotational instability was observed. There were no significant differences in outcome or joint stability between surgically and non-surgically treated apple- bite fracture.

**Table 2:** Final outcome

Final Outcome	
**Complete bone healing (%)**	100
**Return to sports (months)**	8.3 ± 3
**Return to work (months)**	4.9 ± 2
**Lysholm Score**	84 ± 13
**International Knee Documentation Committee** (**IKDC) Score (%)**	77.9 ± 11
**Knee Injury and Osteoarthritis Outcome Score** (**KOOS)**	81 ± 14
**Tegener activity scale (TAS)**	5 ± 1
**Pain (Numeric rating scale (NRS))**	2 ± 2
**Residual Knee anteroposterior (AP)-Instability**	
1° 2° 3°	6 (37.5%) 0 0
**Residual Rotational Instability**	0
**Complications**	0
**Surgical revisions**	0

## Discussion

In our study, we analyzed the outcome of surgical treatment of combined injuries involving bony avulsion of the ACL with a concomitant impression fracture (apple-bite fracture) of the posterolateral tibial plateau. To the best of our knowledge, we present the largest study group of these rare sub- entity of tibial plateau fractures. We could demonstrate that arthroscopic treatment of these specific osteoligamentous injuries is successfully possible. An anatomical reconstruction as well as a good clinical outcome with sufficient joint stability can be achieved. Most patients reach a “restitution ad integrum” in relation to work and sport. These results are underlined by studies on single eminence fractures, which mostly describe less pain, shorter hospital stay, fewer nonunions and less soft tissue compromise with an arthroscopic- than with an open procedure, regardless of the fixation method [11–13]. However, our study showed a remarkable higher proportion of residual ACL instabilities (grade 1) in the transosseous suture group (3/4 patients) than in the screw fixation group (3/12), although the patient group size was too small for a statistically valid conclusion. Nevertheless, we consider it conceivable that screw fixation may be more stable than transosseous sutures, especially in cases of large eminence fragments. In multi-fracture situations of the eminence where the fragments are too small for screw fixation, suture pull-out is therefore more likely to be used. Consequently, a more complex fracture situation of the eminence could also be associated with a higher rate of residual laxity due to the more difficult healing. In contrast, with regard to residual ACL laxity, there was no indication of an influence of the posterolateral fracture fixation (three patients treated with posterolateral fixation vs. 3 treated non-surgically).

However, the treatment algorithm of posterolateral tibial plateau fractures and especially of the rare osteoligamentous injuries is still of high scientific interest, since the best surgical approach as well as the indication for non-surgical or surgical treatment are still under debate. This is due to its special, “difficult” anatomical location and the described, possible (multidimensional) joint instability, but not least due to the very heterogeneous fracture morphologies.

First, the visualization of the posterior segments is known to be limited [3, 4] and therefore the anatomic reduction quite difficult. This is supported by the work of Meulenkamp et al., who found an insufficient reduction with an articular step-off greater than 2 mm in 32% of all tibial plateau fractures [6]. The posterior quadrants were particularly affected [6]. As a result, the current literature has especially dealt with the improvement of visualization in the treatment of posterior fractures of the tibial plateau [14–18]. An arthroscopic assistance seems to support the visualization of the posterolateral corner and helps to restore the joint line anatomically without residual steps and good functional outcomes [19–21]. Alternative procedures include fracturoscopy and extended approaches according to Krause t al. [3, 16, 22] and Behrendt et al. [23, 24], which might be necessary in the treatment of multifragmentary type C fractures with the involvement of the posterolateral corner. However, these are significantly more invasive and, in our opinion, not necessary in case of the described apple-bite fractures.

On the one hand, there is more or less consensus about the indication for surgery regarding the fracture’s step-off height. The tolerable limit for a residual step-off height appears to be approximately 2 mm [25–32]. For example, a clinical study by Singleton et al. analyzed the outcomes of 41 patients after tibial plateau fracture in terms of articular congruity. The intraarticular step was measured on coronal plane tomograms, so that posterior fractures could not be evaluated. The authors found that patients with an intraarticular step < 2.5 mm had a better functional outcome in terms of range of motion and Oxford, Iowa and KOOS scores [25]. Parkkinen et al. also tried to identify predictors of early osteoarthritis following lateral tibia plateau fractures as a function of the postoperative mechanical axis and articular congruity. In summary, a valgus malalignment greater than 5° and an articular depression greater than 2 mm led to advanced osteoarthritis, whereas a normal mechanical axis or a depression less than 2 mm did not [27]. Few studies have examined the topic of articular congruity biomechanically [29, 32, 33]. Bai et al. were among the first to demonstrate the problem of increasing contact pressures in the knee joint with increasing articular step-offs, using the example of lateral split fractures in a biomechanical cadaveric study [29]. Contact stress and contact area as well as joint axis were analyzed in 6 human fresh frozen cadaveric knees at 0° and 30° flexion in a static setup. At a 6-mm step-off with 0° knee flexion, the average contact pressures and maximum contact pressures increased an average of 208% and 97%, respectively, and the contact area decreased an average of 33% (*p* < 0.05) [29]. Walter et al. [34] also analyzed the dissipated energy as a parameter of friction and reduction accuracy in lateral tibial split fractures with 2-mm step- and gap-off by studying 6 human cadaveric knees under cyclic loading in a robotic system. They found that a step-down of 2 mm led to a doubling of the dissipated energy, whereas the step-up even tripled. However, a 2-mm gap also led to a statistically significant increase in the dissipated energy, even if it was less than a step [34].

One the other hand, recent studies demonstrate that not only an intraarticular step-off is essential for the clinical outcome and possible development of osteoarthritis, but also the location of the fracture and concomitant ligamentous injuries might play an important role with regard to joint stability [35–38]. The analyzed posterolateral impressions are caused by flexion-, internal rotation- and valgus stress [39]. In addition to the posterolateral impression, tension stress on the anteromedial and posterolateral structures is the result of this three-dimensional stress, which in turn can lead to accompanying lesions of the ACL, anterolateral ligament (ALL), anteromedial structures and the posterolateral corner [40]. Therefore, in addition to the reconstruction of the posterolateral impression, the treatment of possible accompanying ligament injuries plays an important role to restore ligamentous stability. Moreover, a remaining or untreated posterolateral impression itself can on the one hand lead to a progressive sagittal malalignment with therefore a higher tibial slope, which in turn might lead to a failure of the ACL reconstruction [41] or meniscal root tear [42]^,^ [43]. There are also indications in the literature that posterolateral tibial impressions cause a persistent translational and anterolateral rotational instability in combination with ACL deficiency [36–38].

In the past, different studies have investigated the special entity of apple bite fractures. Most notably, Menzdorf et al. described on of the largest collectives of apple bite fractures with ACL injuries to date [44]. They demonstrated the first short-term results of 20 patients with posterolateral tibial plateau fracture with accompanying ACL tear. One of these patients had a bony ACL tear, which corresponds to our collective. The patients reached a subjective IKDC score of 79,15 +/- 6,07, which is similar to our study results. A graft failure of the ACL was not mentioned. In summary, the study provided valuable short-term functional results and a very differentiated view of these heterogeneous fracture patterns. In addition to the clinical results, Menzdorf et al. namely describe a treatment algorithm that includes not only the joint level but also the positional relationship to the lateral meniscus. The authors conclude that posterolateral fractures with a joint step greater than 2 mm and a lack of more than 50% of the posterior horn of the lateral meniscus should be addressed [44]. This can usually be achieved arthroscopically, as shown in our study. This treatment algorithm is very helpful in determining the indication for surgery and is a further development of the description of Bernholt et al. who already described morphologic variants of posterolateral tibial plateau fractures with accompanying ACL tears [45]. Korthaus et al. were also able to demonstrate the frequent occurrence of posterolateral tibial plateau fractures in the context of knee dislocations in a recent study [35]. This once again demonstrates the close connection between ligament injuries and posterolateral fractures due to the multidimensional trauma mechanism, which, in our view, also necessitates osteoligamentary reconstruction in the aforementioned indications.

In our study, we indicated the reconstruction of the posterolateral corner accordingly to a 2 mm step dislocation and the size of the affected joint surface. In patients with unstable eminence fractures and small posterolateral rim fractures < 50% of the lateral meniscus only the eminence fracture was fixed. At this point, it must be noted that our work, analogous to the study by Menzdorf et al., does not provide exact threshold values for surgical or nonsurgical treatment due to the small sample size and the heterogeneous fracture morphologies. However, since we could also demonstrate sufficient functional results and joint stability with an analogous surgical procedure, the treatment algorithm for apple bite fractures with accompanying ACL tears according to Menzdorf et al. [44] provides a successful and sensible approach.

Despite attempts to ensure reliability, there are different limitations to our study. First, the retrospective study design led to an inhomogeneous follow-up period among the patients, which in turn could possibly lead to bias in clinical outcome scores. In addition, the study group is rather small in absolute terms, but in relation to the very rare combined fracture incidence, it is the largest described to date. The study also exhibits heterogeneity regarding the fixation method used for bony ACL lesions. This could potentially influence the results, particularly concerning residual ACL laxity, as already mentioned in the discussion. Furthermore, we provide short- to midterm clinical and radiographic follow-ups as part of the study; therefore, a general statement regarding the posttraumatic osteoarthritis rate and long-term joint stability is not possible for all patients. Nevertheless, our study provides new, valuable clinical results and supports a very soft tissue-sparing, minimally invasive surgical procedure for the treatment of these rare osteoligamentous injuries.

## Conclusion

Arthroscopically assisted reduction and fixation of combined bony ACL tears and posterolateral tibial plateau fractures as a minimally invasive treatment procedure results in very good functional and clinical outcomes with sufficient joint stability. The excellent visualization of the joint surface supports an anatomical reconstruction of the fracture and the joint surface.

## Data Availability

No datasets were generated or analysed during the current study.
